# Sex-Related Differences among Adults with Hypertrophic Obstructive Cardiomyopathy Undergoing Transcoronary Ablation of Septal Hypertrophy

**DOI:** 10.3390/jcm12083024

**Published:** 2023-04-21

**Authors:** Emyal Alyaydin, Julia Kirsten Vogel, Peter Luedike, Tienush Rassaf, Rolf Alexander Jánosi, Maria Papathanasiou

**Affiliations:** Department of Cardiology and Vascular Medicine, West German Heart and Vascular Center, University Hospital Essen, Hufelanstrasse 55, 45147 Essen, Germany

**Keywords:** hypertrophic cardiomyopathy, transcoronary ablation of septal hypertrophy, sex-related differences, outcome, prognosis

## Abstract

(1) Background: The transcoronary ablation of septal hypertrophy (TASH) is an established therapy for hypertrophic obstructive cardiomyopathy (HOCM). Previous studies on this topic are characterised by a consistent male predominance and show a worse prognosis in females. (2) Methods: This study is a retrospective analysis of all TASH procedures conducted between 2006 and 2021 at a tertiary academic centre. A solution of 75 µm microspheres (Embozene^®^, Boston Scientific, Marlborough, MA, USA) was used as an embolising agent. The outcomes of interest were left ventricular outflow tract (LVOT) gradient reduction and symptom improvement among males vs. that among females. Secondarily, we analysed the sex-related differences in procedural safety outcomes and mortality. (3) Results: The study population consisted of 76 patients, with a median age of 61 years. Females comprised 57% of the cohort. We observed no sex-related differences in the baseline LVOT gradients at rest or under provocation (*p* = 0.560 and *p* = 0.208, respectively). Females were significantly older at the time of the procedure (*p* < 0.001), had lower tricuspid annular systolic excursion (TAPSE) (*p* = 0.009), presented a worse clinical status according to the NYHA functional classification (for NYHA ≥ 3, *p* < 0.001), and were more often on diuretics (*p* < 0.001). We did not observe sex-related differences in absolute gradient reduction at rest (*p* = 0.147) and under provocation (*p* = 0.709). There was a reduction in the NYHA class by a median value of 1 (*p* = 0.636) at follow-up for both sexes. Postprocedural access site complications were documented in four cases (two of which concerned females), and complete atrioventricular block was noted in five patients (three of which concerned females). The 10-year survival rates were comparable between the sexes (85% in females and 88% in males). The female sex was not associated with enhanced mortality according to multivariate analysis after adjusting for the confounding variables (HR 0.94; 95% CI 0.376–2.350; *p* = 0.895), but we observed age-related differences in long-term mortality (HR 1.035; 95% CI 1.007–1.063; *p* = 0.015). (4) Conclusions: TASH is safe and effective in both sexes, irrespective of their clinical differences. Women present at an advanced age and with more severe symptoms. An advanced age at the time of the intervention is an independent predictor of mortality.

## 1. Introduction

The current level of awareness of cardiomyopathies in women is insufficient, leading to under-recognition, delayed identification, and treatment disparities compared to their male counterparts [1]. Studies reporting sex-related differences in the outcomes of hypertrophic obstructive cardiomyopathy (HOCM) are characterised by male predominance, and the results regarding disease manifestation and outcomes remain contradictory [2,3,4,5,6]. While some results indicate more pronounced obstruction, higher prevalence of heart failure, and worse cardiopulmonary exercise tolerance, which is associated with inferior survival in women [2,3,4], in other cases, the sex-related differences in the disease phenotype are considered to have no impact on mortality [6]. Irrespective of the patient’s sex, the transcoronary ablation of septal hypertrophy (TASH) and surgical septal myectomy (SSM) are the therapies of choice for patients resistant to pharmacological treatment [7,8,9,10].

This study aimed to investigate the effect of sex-related differences in clinical phenotypes on procedural outcomes in patients undergoing TASH using microspheres.

## 2. Materials and Methods

### 2.1. Study Population and Design

**Figure 1:**
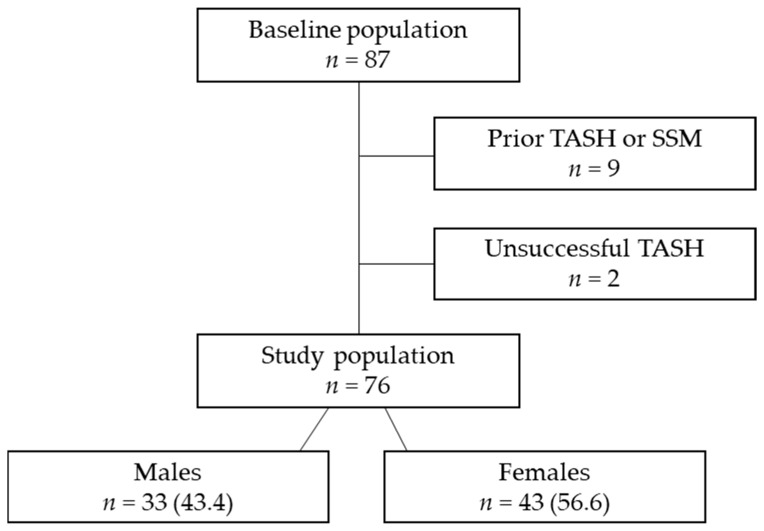
Derivation of the analytic cohort. TASH—transcoronary ablation of septal hypertrophy using alcohol; SSM—surgical septal myectomy. Data are presented as *n* (%).

This is a retrospective analysis of sex differences in the clinical characteristics and outcomes of adult patients who have undergone TASH at the West German Heart and Vascular Centre. Data were retrieved from the patients’ electronic health records. We included all subjects who had undergone TASH over the last 15 years (April 2006 to July 2021). Figure 1 depicts the derivation of the study cohort. Incomplete follow-up, age < 18 years, and prior interventional or surgical therapy for septal reduction were the study’s exclusion criteria. The study population comprised 87 patients. Nine patients were excluded because they underwent prior TASH or SSM before referral to our centre. Additionally, two patients were excluded due to unfavourable anatomy of the septal perforators, rendering the intervention unsuccessful. The remaining 76 patients were stratified into two groups according to their sex (males: *n* =33, 43%; females: *n* = 43, 57%). Indications for TASH were LVOT gradient at rest ≥ 30 mmHg and/or with Valsalva manoeuvre ≥ 50 mmHg in combination with clinical symptoms (dyspnoea, syncope, cardiac arrest, and arrhythmia). The predefined inclusion criteria were age ≥ 18 years at the time of intervention and complete assessment, including initial investigations, periprocedural data, and follow-up results encompassing clinical, echocardiographic, and laboratory data.

The primary endpoint was the difference in peak left ventricular outflow tract gradients (Δ left ventricular outflow tract (LVOT)) among men and women, considering the echocardiographically derived peak gradients before the intervention and at the first scheduled follow-up. Secondary endpoints were major adverse cardiovascular events, including stroke and cardiovascular death, and high-degree atrioventricular (AV) block, access site complications, arrhythmias, the need for redo-procedures, and long-term mortality with regard to patient sex. 

### 2.2. TASH Protocol and Non-Invasive Investigations

LVOT gradients were estimated using transthoracic echocardiography at rest and under Valsalva provocation before and after the intervention. The interventricular septal diameter (IVSd), systolic anterior motion (SAM) of the mitral valve, and the left atrial (LA) diameter were evaluated in M-mode. The biplane Simpson’s method was used to estimate left ventricular ejection fraction. Daily echocardiographic monitoring was performed after a successful procedure. According to a predefined institutional protocol, patients were scheduled for a follow-up assessment three to six months after the TASH procedure. Additionally, blood tests were performed concerning coagulation, complete blood count, liver and renal function, and N-terminal prohormone of B-type natriuretic peptide (NT-proBNP). The cardiac enzymes were measured at six-hour intervals after TASH to determine the peak value and further assessed until reaching 50% of the peak values.

All septal reduction procedures were conducted by embolization of the septal perforator of interest using a solution of 75 µm microspheres (Embozene^®^, Boston Scientific) [11]. This medium is implemented for the treatment of hyper-vascular tumours. The higher viscosity and non-resorbable nature of the microspheres offer potential advantages over alcohol. Since 2005, microspheres have been the medium of choice for TASH procedures at our centre [12]. All procedures were performed by experienced professionals. Over the fifteen years encompassed by our study, four interventional cardiologists mastered the TASH technique, and the procedures were conducted in the presence of at least two of them. All interventions were performed via the femoral approach. A temporary pacemaker lead was advanced using a venous sheath. Simultaneous LVOT gradient assessment was conducted by inserting two pigtail catheters into the left ventricle (LV) and the ascending aorta. After wiring the target septal branch, an over-the-wire balloon was used to occlude the vessel. Subsequently, the application of microspheres was performed under fluoroscopic guidance until angiographic evidence of vessel occlusion was obtained. After the procedure, patients were admitted to the intermediate care unit for at least 24 h. 

### 2.3. Statistical Analysis

All statistical analyses were performed using IBM SPSS Statistics software (version 29). The Shapiro–Wilk test was used to determine the normality of the data distribution. Continuous variables are given as mean ± standard deviation (mean ± SD) if normally distributed and median and interquartile range (IQR) if skewed. Normally distributed data were further assessed using Student’s t-test. Otherwise, the non-parametric Mann–Whitney U test was used. Categorical variables are presented as numbers (percentages) and were analysed using the chi-square test. Assessment of factors associated with long-term mortality in patients undergoing TASH was performed using Cox regression analysis with stepwise backward selection. Variables for which *p* < 0.10 were included in the final model. Statistical significance was set at *p* < 0.05.

## 3. Results

### 3.1. Baseline Characteristics

The study cohort comprised 76 patients. Females constituted more than half of the population (*n* = 43, 57%) and were older at admission for TASH compared to their male counterparts (median age 68 years in females vs. 54 years in males; *p* < 0.001) (Table 1). Severe dyspnoea was the leading symptom upon initial presentation (NYHA ≥ 3 in 86% of the patients), for which there were significant differences between both sexes (93% in females and 76% in males; *p* = 0.049). A fifth of the patients had syncope, for which there no sex-related differences (24% in males and 14% in females; *p* = 0.371). Previous cardiac arrest or sustained ventricular tachycardia with hemodynamic instability were reported in 4% of the population (6% in males and 2% in females; *p* = 0.576). One fifth of the study population had undergone previous device implantation, for which there was a non-significant predominance of implantable cardiac defibrillators (ICDs) in males (*n* = 5, 12% in females vs. *n* = 8, 24% in males; *p* = 0.219). Hypertension was the most common comorbidity irrespective of patient sex (*n* = 37, 86% in females vs. *n* = 28, 85% in males; *p* = 1.000). Diabetes and atrial fibrillation had an overall prevalence of 17%, for which there were no sex-related differences (*p* = 0.766 and *p* = 1.000, respectively). We observed a non-significant difference in the rate of coronary artery disease, with 35% of females affected compared to 21% of males (*p* = 0.214). Inter- or intraventricular conduction disturbances not requiring pacemaker implantation were observed in less than 10% of the overall cohort, for which there were no sex-related differences (Table 1).

The median left ventricular ejection fraction (LVEF) was 61.0 [11.0]%. The median LVOT gradients at rest (42 [32.0] mmHg in females vs. 40 [28.0] mmHg in males; *p* = 0.560) and during Valsalva provocation (85 [60.0] mmHg in females vs. 95 [75.0] mmHg in males; *p* = 0.208) were comparable in both sexes. There was no sex-related difference in the rate of systolic anterior motion (SAM) of the mitral valve (*n* = 33, 77%, in females vs. *n* = 29, 88%, in males; *p* = 0.248). Females presented with lower tricuspid annular systolic excursion (TAPSE) (19 mm in females vs. 21 mm in males; *p* = 0.009). As depicted in Table 1, beta-blockers were the most utilised agent in the overall population irrespective of sex (*n* = 62, 82%), whereas verapamil was the drug of second choice (*n* = 16, 21%). Significantly more females were on diuretics (*n* = 33, 77%, for females vs. *n* = 12, 36%, for males; *p* < 0.001). NT-proBNP levels were non-significantly elevated in females compared to their male counterparts (431 [1142.0] pg/mL in females vs. 318 [562.0] pg/mL in males; *p*= 0.099). Additionally, more than half of the patients were taking antiplatelet agents (*n* = 42, 55%), whereas oral anticoagulation was implemented in 16% of the population (Table 1).

### 3.2. Periprocedural Outcomes

As shown in Table 2, there were no significant differences in the volume of microspheres used to occlude the septal perforator of interest (2 [2.0] mL in females vs. 3 [3.0] mL in males; *p* = 0.306). 

We observed no differences in the maximal postprocedural levels of Troponin I levels between the study groups (2659 [2889.0] ng/L in females vs. 2311 [2908.0] ng/L in males; *p* = 0.506). There were no significant differences in the incidence of postprocedural complete atrioventricular (AV) block, left bundle branch block (LBBB), or right bundle branch block (RBBB) (Table 2). When considering AV and interventricular conduction disturbances as a composite entity, the overall incidence of conduction disturbances was 14% in females and 9% in males (*p* = 0.518). We did not observe an impact of age on the incidence of complete AV block after TASH in our cohort of patients (OR 1.02; 95% CI 0.957–1.084; *p* = 0.564). Access site complications were observed in four patients (*n* = 2 in females; *p* = 0.786). Intraprocedural death occurred in one case of urgent TASH, which was performed as a rescue procedure following haemodynamic instability immediately after the deployment of an aortic valve prosthesis during transcatheter aortic valve replacement (TAVR) with a multifactorial aetiology. This is a rare indication, as previously mentioned in the medical literature [13,14]. We did not observe any periprocedural stroke events or arrhythmias. 

As depicted in Table 3, the routine follow-up assessment of the patients after a median period of 4 [8.0] months revealed a significant reduction in LVOT gradients at rest and after Valsalva provocation (*p* < 0.001 for both sexes), without differences between the study groups (females vs. males; *p* = 0.147 at rest and *p* = 0.709 under provocation). LVOT gradient < 30 mmHg at rest was achieved in 88% of the patients, without differences between the sexes (*p* = 0.948). Provoked gradient < 50 mmHg was reported in 70% of males and 79% of females (*p* = 0.353). Furthermore, we observed a significant reduction in the prevalence of severe dyspnoea according to the New York Heart Association (NYHA) functional classification (NYHA ≥ 3: 76% at baseline assessment to 3% following TASH in males, *p* < 0.001, and 93% to 5% in females, *p* < 0.001) irrespective of patients’ sex (*p* = 0.636). There was a significant reduction in NT-proBNP levels at follow-up in females (431 pg/mL to 281 pg/mL; *p* = 0.009) compared with males (318 pg/mL to 189 pg/mL; *p* = 0.073) (Table 3).

### 3.3. Long-Term Follow-up

The 10-year survival rates were comparable in both sexes (85% in females and 88% in males). The female sex was not associated with enhanced mortality according to multivariate analysis after adjustment for the confounding variables (TAPSE, use of diuretics, left atrial diameter, age at TASH, and NYHA class) (HR 0.94; 95% CI 0.38–2.35; *p* = 0.895), but there were age-related differences in mortality (HR 1.035; 95% CI 1.007–1.063; *p* = 0.015) (Table 4).

We did not observe a significant impact of the estimated gradient measures, family history of SCD, a previous pacemaker or ICD implantation, LVEF, IVSd ≥ 20 mm, or sex-related differences on the primary outcome measures. The male sex was associated with a non-significantly elevated rate of redo procedures at follow-up (25% in males vs. 13.6% in females; *p* = 0.445). 

## 4. Discussion

In contrast to previous studies, our cohort is characterised by female predominance [9,10,11]. As we report on the results of a tertiary referral centre with well-established experience in treating patients with cardiomyopathies, the study cohort may have included some high-risk individuals. For a disease considered equally prevalent among males and females, the female predominance of patients referred for TASH may be due to the more pronounced disease manifestations in women, resistance to conservative treatment, more obstructive physiology, or even lack of disease awareness among females [2,3,4]. A potential indicator in favour of the latter is that females were significantly older at admission for TASH, with a median age difference of 14 years. A recent analysis of registry data reported a similar age distribution [5]. The significantly higher rate of severe dyspnoea in females is an alarming sign of a potential failure to recognise the isolated disease symptoms and comorbidities, contributing to the more advanced impairment of their physical capacity. The higher rate of diuretic treatment also suggests an advanced disease stage with signs of congestion. This is in accordance with previous studies reporting a higher risk of developing severe heart failure in females with HOCM [2,15]. The more pronounced disease manifestation in females may also be due to the higher burden of sarcomere variants [15].

We did not observe any differences in the prevalence of a positive family history of sudden cardiac death or previous syncope. There was a non-significant male predominance in the cardiac defibrillator implantation rate. Previous studies have reported a higher risk of exercise-induced non-sustained and sustained ventricular tachycardias in males with HCM compared to females [16]. Additionally, females with HOCM are known to have a lower left ventricular mass, fewer fibrotic changes, and a lower rate of electrocardiographic abnormalities [17,18]. Due to the retrospective nature of this analysis, we do not have sufficient data regarding disease history or exercise-related electrocardiographic abnormalities. 

The median LVEF was within the normal range in our patient population, and we observed no significant differences in the LVOT gradients at rest or under Valsalva provocation. The larger diameter of the left atrium in females may indicate more pronounced diastolic dysfunction and elevated filling pressure due to disease progression [19]. Combined with the finding of a lower TAPSE, this may be a warning sign of worsening heart failure. Recent studies based on a magnetic-resonance-guided assessment of the biventricular function in HOCM found that right ventricular function declines prior to that of the LV as assessed by LVEF during the course of disease [20]. Our observations are consistent with those of previous studies reporting that heart failure is the most common feature leading to the diagnosis of HCM in females. In contrast, in males, diagnosis often follows a routine medical assessment with the identification of an abnormal electrocardiogram or heart murmur [6]. There are still relevant sex disparities in terms of the percentage of subjects undergoing routine health risk assessments, with males more often being the focus of preventive care [21].

Despite sex-related differences in the baseline characteristics, the TASH procedure was associated with a significant reduction in LVOT gradients at rest and under provocation, without differences between the study groups. Relief from LVOT obstruction led to a significant symptomatic improvement irrespective of sex. Additionally, there was a significant reduction in NT-proBNP in females, which was potentially due to advanced disease and more pronounced congestion. Advanced age has been previously reported to be associated with a higher rate of conduction disturbance following TASH using alcohol [22]. We did not observe correlation between age and AV block. This may be attributed to the advantages of microspheres over alcohol for septal reduction. On the one hand, the higher viscosity of microspheres may reduce the risk of accidental off-target coronary embolization. Their inability to be resorbed reduces the risk of cardiotoxicity compared with alcohol [23]. The rate of complete AV block in our population was lower than the incidence reported for TASH with alcohol [24]. The volume of microspheres was non-significantly higher in males. The amount of injected medium was much lower than that in TASH using alcohol, where an association between alcohol volume and postprocedural conduction disturbances has been reported [25]. We observed no differences in the rate of access site complications between the sexes, and the overall incidence was lower than that previously reported for TASH using the femoral approach [26]. 

Regarding long-term prognosis, the 10-year survival rates were comparable between the sexes. The female sex was not associated with enhanced mortality in the multivariate analysis after adjusting for the confounding variables, and age was the only independent predictor of mortality. This is in line with the results reported to date, indicating a higher mortality risk at advanced age, which is potentially related to disease progression and higher comorbidity burden of the patients [25]. 

It remains unknown whether there are any sex-related differences in the rates of recurrent LVOT obstruction after TASH using microspheres, an outcome that is not specifically addressed in the current analysis. There were no differences in the rates of redo procedures in our patient population. However, in the era of disease-modifying therapies using myosin inhibitors, it is of profound interest to identify whether sex could be a confounder of outcomes beyond LVOT gradient reduction [27,28]. The female sex is associated with a higher rate of sarcomere mutations [15,21]; potential sex-related differences in the long-term effectiveness of myosin inhibitors remain yet to be investigated.

Our study has inherent limitations associated with its retrospective design. We reported on all-cause mortality, but we did not have data regarding the specific cause of death of all patients. Long-term outcomes of gradient reduction and symptom severity after TASH were not consistently available. The microsphere solution for septal embolization is not directly comparable to alcohol, and its utility is limited because of its higher cost. Compared to radiofrequency ablation, TASH using microspheres is associated with a comparable reduction in LVOT gradients at rest and under provocation [29]. The complete AV block and periprocedural complication rates are higher in radiofrequency ablation, and there are no data regarding the rate of recurrent obstruction. Due to the limited experience and small sample size of studies concerning radiofrequency ablation, this result should be interpreted with caution. Nevertheless, it remains a medium with potential advantages in terms of complication rates.

## 5. Conclusions

Women with HOCM present at a more advanced age, with more severe symptoms and a poor clinical status. TASH using microspheres is safe and effective in both sexes irrespective of their clinical differences. Age was an independent predictor of long-term mortality after TASH regardless of sex.

## Figures and Tables

**Table 1:** Baseline characteristics of the patient population.

Patient Characteristics	Overall Population *n* = 76	Male *n* = 33 (43.4)	Female *n* = 43 (56.6)	*p* Value
1. Demographics				
Age, years	60.5 [26.0]	54.0 [22.0]	68.0 [21.0]	<0.001
BMI, kg/m^2^	28.2 ± 4.8	28.6 ± 4.8	27.9 ± 4.9	0.509
BSA, m^2^	1.9 ± 0.3	2.0 ± 0.3	1.8 ± 0.2	<0.001
NYHA ≥ 3, *n* (%)	65 (85.5)	25 (75.8)	40 (93.0)	0.049
Previous syncope, *n* (%)	14 (18.4)	8 (24.2)	6 (14.0)	0.371
Family history of SCD, *n* (%)	11 (14.5)	6 (18.2)	5 (11.6)	0.517
Previous cardiac arrest or sustained VT, *n* (%)	3 (3.9)	2 (6.1)	1 (2.3)	0.576
ICD, *n* (%)	13 (17.1)	8 (24.2)	5 (11.6)	0.219
Pacemaker, *n* (%)	2 (2.6)	1 (3.0)	1 (2.3)	1.000
CRT, *n* (%)	1 (1.3)	0 (0.0)	1 (2.3)	1.000
2. Comorbidities				
HTN, *n* (%)	65 (85.5)	28 (84.8)	37 (86.0)	1.000
Diabetes mellitus, *n* (%)	13 (17.1)	5 (15.2)	8 (18.6)	0.766
Atrial fibrillation, *n* (%)	13 (17.1)	6 (18.2)	7 (16.3)	1.000
CAD, *n* (%)	22 (28.9)	7 (21.2)	15 (34.9)	0.214
COPD, *n* (%)	6 (7.9)	3 (9.1)	3 (7.0)	1.000
AV block, *n* (%)	5 (6.7)	1 (3.0)	4 (9.3)	0.381
LAFB, *n* (%)	5 (6.6)	3 (9.1)	2 (4.7)	0.647
LBBB, *n* (%)	6 (7.9)	2 (6.1)	4 (9.3)	0.692
RBBB, *n* (%)	6 (7.9)	5 (15.2)	1 (2.3)	0.080
nsVT, *n* (%)	8 (10.5)	3 (9.1)	5 (11.6)	1.000
3. Echocardiography				
LVEF, %	61 [11.0]	63 [14.0]	60 [8.0]	0.113
LVOT gradient at rest, mmHg	41.0 [27.0]	40.0 [28.0]	42.0 [32.0]	0.560
LVOT gradient (Valsalva), mmHg	95.0 [71.0]	95.0 [75.0]	85.0 [60.0]	0.208
SAM, *n* (%)	62 (81.6)	29 (87.9)	33 (76.7)	0.248
MR ≥ 2 grade, *n* (%)	35 (46.1)	16 (48.5)	19 (44.2)	0.817
LA diameter/m^2^, mm	22.0 [4.6]	21.5 [4.7]	23.7 [5.3]	0.040
LAVI/m^2^, mL	38.9 [20.0]	34.8 [4.7]	40.1 [20.5]	0.283
TAPSE, mm	19 [4.0]	21 [5.0]	19 [3.0]	0.009
sPAP > 35 mmHg, *n* (%)	22 (28.9)	9 (27.3)	13 (30.2)	0.805
4. Laboratory results				
NTproBNP, pg/mL	388.0 [737.0]	318.0 [562.0]	430.7 [1142.0]	0.099
Creatinine, mg/dL	1.1 [0.2]	1.1 [0.3]	1.1 [0.2]	0.146
CK, U/L	75.0 [53.5]	77.0 [33.0]	69.0 [67.0]	0.052
TroponinI, ng/L	20.0 [30.0]	20.0 [48.5]	20.0 [30.0]	0.577
Medical treatment				
Betablockers, *n* (%)	62 (81.6)	29 (87.9)	33 (76.7)	0.248
Verapamil, *n* (%)	16 (21.1)	6 (18.2)	10 (23.3)	0.778
Diltiazem, *n* (%)	1 (1.3)	1 (3.1)	0 (0.0)	0.427
Diuretics, *n* (%)	45 (59.2)	12 (36.4)	33 (76.7)	<0.001
NOACs, *n* (%)	4 (5.3)	2 (6.1)	2 (4.7)	1.000
Vitamin K antagonists, *n* (%)	8 (10.5)	4 (12.1)	4 (9.3)	0.721
Antiplatelet agents, *n* (%)	42 (55.3)	17 (51.5)	25 (58.1)	0.644

Data are presented as mean ± (SD), median (IQR), or *n* (%). BMI—Body Mass Index, BSA—body surface area (Du Bois Method), NYHA—New York Heart Association (NYHA) Classification, SCD—sudden cardiac death, VT—ventricular tachycardia, ICD—implantable cardioverter defibrillator, CRT—cardiac resynchronization therapy, HTN—hypertension, CAD—coronary artery disease, COPD—chronic obstructive pulmonary disease, AV block—atrioventricular block, LAFB—left anterior fascicular block, LBBB—left bundle branch block, RBBB—right bundle branch block, nsVT—non-sustained ventricular tachycardia, LVEF—left ventricular ejection fraction, LVOT—left ventricular outflow tract, SAM—systolic anterior motion of the mitral valve, MR—mitral regurgitation, LA—left atrium, LAVI—left atrial volume index, TAPSE—tricuspid annular plane systolic excursion, sPAP—systolic pulmonary artery pressure, NT-proBNP—N-terminal prohormone of brain natriuretic peptide, CK—creatine kinase, and NOACs—novel oral anticoagulants.

**Table 2:** Procedural characteristics and complications.

Peri- and Postprocedural Outcomes	Overall Population *n* = 76	Male *n* = 33	Female *n* = 43	*p* Value
Volume of microspheres, mL	2.0 [2.0]	3.0 [3.0]	2.0 [2.0]	0.306
ICU stay, days	1.0 [0.0]	1.0 [0.0]	1.0 [1.0]	0.133
Hospitalisation, days	12.5 [10.0]	12.0 [8.0]	13.0 [11.0]	0.449
CK max, U/L	926.0 [949.0]	1018.0 [1537.0]	897 [947.0]	0.234
Troponin I, ng/L	2485.0 [2874.0]	2311.0 [2908.0]	2659.0 [2889.0]	0.506
Complications				
AV block, *n* (%)	5 (6.6)	2 (6.1)	3 (7.0)	1.000
New LBBB, *n* (<%)	1 (1.3)	0 (0.0)	1 (2.3)	1.000
New RBBB, *n* (%)	3 (3.9)	1 (3.0)	2 (4.7)	1.000
Access site complications, *n* (%)	4 (5.3)	2 (6.1)	2 (4.7)	1.000
Postprocedural death, *n* (%)	1 (1.3)	1 (3.0)	0 (0.0)	0.434

ICU—intensive care unit, CK—creatine kinase, AV block—atrioventricular block, LBBB—left bundle branch block, and RBBB—right bundle branch block. Data are presented as *n* (%) or median (IQR).

**Table 3:** Short-term outcomes after TASH (initial assessment and first clinical follow-up at 4 [8.0] months).

Characteristics	Males (*n* = 33)			Females (*n* = 43)			Males vs. Females
	Pre-TASH	Post-TASH	*p* Value	Pre-TASH	Post-TASH	*p* Value	*p* Value for Δ
LVOT gradient at rest, mmHg	40 [22.0]	16.0 [14.0]	<0.001	42.0 [32.0]	15.0 [17.0]	<0.001	0.147
Provoked LVOT gradient, mmHg	95.0 [75.0]	25.0 [41.0]	<0.001	85.0 [60.0]	24.0 [42.0]	<0.001	0.709
NT-proBNP, pg/mL	318.0 [562.0]	189.0 [367.0]	0.073	430.7 [1142.0]	281.0 [408.0]	0.009	0.338
NYHA ≥ 3, *n* (%)	25 (75.8)	1 (3.0)	<0.001	40 (93.0)	2 (4.7)	<0.001	0.636
IVSd > 20 mm, *n* (%)	16 (48.5)	4 (12.1)	<0.001	21 (48.8)	3 (7.0)	<0.001	0.583

LVOT—left ventricular outflow tract, NYHA—Ney York Heart Association functional classification, IVSd—Interventricular septum thickness in diastole, and TASH—transcoronary ablation of septal hypertrophy. Data are presented as median (IQR) and *n* (%).

**Table 4:** Multivariate Cox regression analysis.

Characteristics	HR	95% CI	*p* Value
Age at TASH, years	1.035	1.007–1.063	0.015
NYHA ≥ 3	1.256	0.359–4.395	0.722
Female sex	0.940	0.376–2.350	0.895
TAPSE, mm	1.921	0.802–1.057	0.240
Diuretics	1.703	0.707–4.101	0.235
LA diameter, mm	1.007	0.974–1.040	0.697

TASH—transcoronary ablation of septal hypertrophy, NYHA—New York Heart Association, TAPSE—Tricuspid annular systolic excursion, and LA dimeter—left atrial diameter in parasternal long-axis view.

## Data Availability

The data presented in this study are available on reasonable request from the corresponding author. The data are not publicly available due to data confidentiality regulation.
